# Possible bi-directional link between ET_A_ receptors and protein kinase C in rat blood vessels

**DOI:** 10.1155/S096293519500010X

**Published:** 1995-01

**Authors:** A. M. Northover, B. J. Northover

**Affiliations:** Department of Pharmaceutical Sciences School of Applied Sciences De Montfort University Leicester LE1 9BH UK

## Abstract

Possible links have been investigated between activation of protein kinase C (PKC) and endothelin (ET) production by small blood vessels. Perfusion pressures were recorded from rat isolated mesenteric artery, with or without the small intestine attached, before and after addition to the perfusate of either ET-1, ET-3 or the PKC activator 12-deoxyphorbol 13-phenylacetate (DOPPA). Rises in perfusion pressure in response to ET-1 (10^−8^ M)or DOPPA (10^−6^ M) were reduced significantly by pre-treatment with either the ET_A_ receptor antagonist PD151242 (10^−6^ M) or the PKC inhibitor Ro 31-8220 (10^−6^ M). ET-3 (10^−8^ M) had a significant, albeit small, effect only when the gut was still attached to the mesentery. Inthis latter preparation ET-1 and DOPPA increased the permeability of villi microvessels to colloidal carbon in the perfusate. This effect of DOPPA was reduced by pre-treatment with either PD151242 or Ro 31-8220, but the effects of ET-1 were reduced significantly only by Ro 31-8220. ET-3 (10^−8^ M) was without effect. The results suggest a possible bi-directional link between ET_A_ receptors and PKC in the intestinal vasculature.


Research Paper

Mediators of Inflammation 4, 55–59 (1995)

### Possible bi-directional link between $ET_A$ receptors and protein kinase C in rat blood vessels

A. M. Northover[CA] and B. J. Northover

Department of Pharmaceutical Sciences, School of Applied Sciences, De Montfort University, Leicester LE1 9BH, UK

[CA] Corresponding Author

**Possible links have been investigated between activation of protein kinase C (PKC) and endothelin (ET) production by small blood vessels. Perfusion pressures were recorded from rat isolated mesenteric artery, with or without the small intestine attached, before and after addition to the perfusate of either ET-1, ET-3 or the PKC activator 12-deoxyphorbol 13-phenylacetate (DOPPA). Rises in perfusion pressure in response to ET-1 ($10^{-8}$ M) or DOPPA ($10^{-6}$ M) were reduced significantly by pre-treatment with either the $ET_A$ receptor antagonist PD151242 ($10^{-6}$ M) or the PKC inhibitor Ro 31-8220 ($10^{-6}$ M). ET-3 ($10^{-8}$ M) had a significant, albeit small, effect only when the gut was still attached to the mesentery. In this latter preparation ET-1 and DOPPA increased the permeability of villi microvessels to colloidal carbon in the perfusate. This effect of DOPPA was reduced by pre-treatment with either PD151242 or Ro 31-8220, but the effects of ET-1 were reduced significantly only by Ro 31-8220. ET-3 ($10^{-8}$ M) was without effect. The results suggest a possible bi-directional link between $ET_A$ receptors and PKC in the intestinal vasculature.**

**Key words:** Colloidal carbon, 12-Deoxyphorbol 13-phenylacetate, Endothelins, Microvascular permeability, PD151242, Protein kinase C, Ro 31-8220, Vasoconstriction

### Introduction

Endothelin (ET) isolated from porcine[1] and from human[2] endothelial cells (ECs) was shown to consist of three distinct isopeptides, ET-1, ET-2 and ET-3.[3] The release of ET-1 from ECs in response to stimulation by angiotensin or vasopressin,[4] or to shear stress[5] or to cyclical strain[6] has been associated with the activation of protein kinase C (PKC). In addition, it has been suggested recently that the release of ETs,[7] or other vasoconstrictor substances,[8] may be responsible for the slowly developing vasoconstriction elicited by phorbol esters in rat isolated mesenteric arterioles. The present work was undertaken to seek evidence regarding the possible involvement of ET in this experimental situation, since phorbol esters are well known to activate PKC[9,10] and one such has been shown to up-regulate the mRNA for preproendothelin-1 in cultured ECs.[11,12] Phorbol esters have also been shown to increase the permeability to injected colloidal carbon of microvessels in rat small intestinal villi *in vitro*.[7,13] Thus, the effects of ET-1, ET-3 and 12-deoxyphorbol 13-phenylacetate (DOPPA) on perfusion pressure in rat mesenteric arterioles and on the permeability of microvessels to colloidal carbon in rat villi have been compared. In addition, the effects of pre-treatment with PD151242, an $ET_A$ receptor antagonist,[14] and Ro 31-8220, a specific PKC inhibitor,[15] prior to exposure to ET-1 or DOPPA have been examined.

### Methods

The two techniques used in the present experiments have been described in detail elsewhere.[7,8,16] For both preparations rats weighing 350–500 g were killed by inhalation of chloroform vapour, and the anterior mesenteric artery then cannulated. Subsequently, the mesentery, with or without the small intestine attached to it, was removed to an organ bath containing 50 ml of a physiological salt solution (PSS) at 37°C. The blood vessels were flushed with fresh PSS from a reservoir at 37°C via a roller pump until free of visible blood, and then one or other of the following two procedures was used.

*Isolated mesentery:* The rate of flow of perfusate was set to give a perfusion pressure of approximately 35 mmHg. The volumes of PSS in the organ bath and in the reservoir were then adjusted to 50 ml in each case, and re-circulation of PSS commenced. When appropriate, PD151242 or Ro 31-8220 was added to the reservoir to give the required concentration. After 10 min of perfusion in this way, the perfusion pressure was noted and then ET-1 or ET-3 or DOPPA was added to the reservoir. Perfusion pressures were recorded at 1 min intervals for the next 15 min. Taking the perfusion pressure at time zero as 0, the rises during the 15 min agonist treatment were calculated. The PSS had the following composition (mM): NaCl 138, KCl 5, $NaHCO_3$ 10.1, $MgCl_2$ 1.06, $NaH_2PO_4$

© 1995 Rapid Communications of Oxford Ltd

0.416, $CaCl_2$ 2, glucose 10 and pH 7.4, and was gassed throughout with 95% $O_2$ and 5% $CO_2$.

*Mesentery with small intestine attached:* The method was similar to that described above except that the rate of flow of perfusate was set at 10 ml/min before re-circulation of perfusate began, and gelatin (2%) was present in the PSS throughout to reduce oedema formation in the gut. After 10 min of re-circulation of the gelatin-containing perfusate (GPSS), with or without added PD151242 or Ro 31-8220, the perfusion pressure was noted and either ET-1 or ET-3 or DOPPA was added. Perfusion pressures at 1 min intervals were recorded for 5 min, after which fresh GPSS was perfused for 1 min, followed by a bolus of 0.5 ml colloidal carbon suspension injected near the cannula.[16] After a further 5 min period of perfusion with GPSS, 2 ml rat washed red blood cells were injected to provide a visual check that vessels in the gut wall were patent. Then the preparation was removed from the organ bath and six pieces of small intestine, each about 4 cm long, were cut, starting from the caecal end. Each was flushed with normal saline, opened lengthways and stapled to a xylene-resistant coverslip. After fixing in formal saline, dehydrating in graded alcohols and clearing in xylene overnight, the specimens were mounted, mucosal surface uppermost, in DPX mountant. Five areas per coverslip were photographed using Ilford FP4 black and white film at ×100 magnification. Negative micrographs were then subjected to image analysis, using a Seescan system (Cambridge, UK), to assess the amount of colloidal carbon trapped in the villi microvessels, as described in detail earlier.[16] A mean value for each piece of small intestine was calculated and used to give a mean value for each animal. Mean values for each treatment group were then compared statistically. The mean rises in perfusion pressure recorded at the end of the 5 min period of ET-1, ET-3 or DOPPA treatment (taking that at time zero as 0) were also compared.

*Statistical analysis:* Bonferroni's test for comparing more than one treatment group with a single control group was used.

*Chemicals used:* ET-1 and ET-3 were obtained from Sigma Chemical Co., Poole, UK; DOPPA was obtained from Calbiochem-Novachem, Nottingham, UK; PD151242 was a gift from Dr A. Doherty, Parke Davis, Ann Arbor, MI, USA; Ro 31-8220 was a gift from Dr G. Lawton, Roche Products, Welwyn Garden City, UK; DPX mountant was obtained from BDH, Poole, UK; gelatin was obtained from Thornton Ross, Huddersfield, UK; Thermanox[R] xylene-resistant coverslips were obtained from Nunc Inc., Naperville, IL, USA; and colloidal carbon (Gunther Wagner, batch C11/1431a) was obtained from Pelikan Inks, Hanover, Germany. DOPPA and Ro 31-8220 were dissolved in dimethyl sulphoxide, which, in the concentration used, was biologically inert; ET-1, ET-3 and PD151242 were dissolved in water, the latter with the aid of a little $Na_2CO_3$. The dose volume used was 0.2 ml.

### Results

In the isolated mesentery without small intestine ET-1 ($10^{-8}$ M) caused a very marked, but slow rise in perfusion pressure, which reached a peak after 13 min of perfusion (Fig. 1). The response was completely abolished by pre-treatment with PD151242 ($10^{-6}$ M) and significantly reduced by pre-treatment with Ro 31-8220 ($10^{-6}$ M). DOPPA($10^{-6}$ M) also caused a gradual rise in perfusion pressure, confirming results from earlier work.[8] This response was significantly reduced by pre-treatment with PD151242, whereas pre-treatment with Ro 31-8220 was shown previously to abolish the effect.[8] Perfusion of the mesenteric arterioles with ET-1 ($10^{-9}$ M) generated a maximum rise of only 6.5 ± 2.6 mmHg. Perfusion with PSS alone (controls), or ET-3 ($10^{-8}$ M), PD151242 alone or Ro 31-8220 alone had no effect on perfusion pressure over a 15 min period.

Perfusion of the vasculature in the isolated mesentery with its small intestine still attached

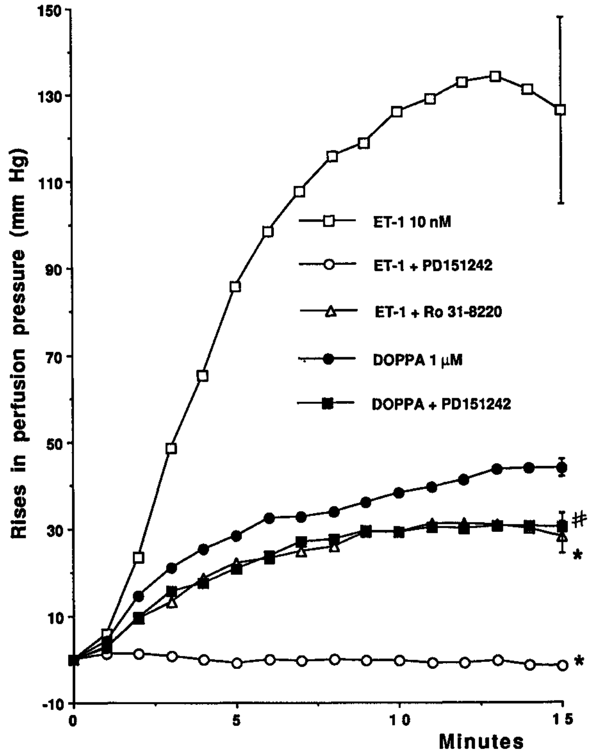

FIG. 1. Changes in perfusion pressure in arterioles of rat isolated mesentery during perfusion with ET-1 ($10^{-8}$ M) or DOPPA ($10^{-6}$ M) in the presence or absence of PD151242 ($10^{-6}$ M) or Ro 31-8220 ($10^{-6}$ M). *A significant difference exists compared with ET-1 alone, or #compared with DOPPA alone ($p < 0.05$, Bonferroni's test). Data points (± S.E.M.) are derived from three to five observations.

yielded results which were somewhat different from those obtained with the mesentery alone. Perfusion with GPSS containing either ET-1 ($10^{-8}$ M) or DOPPA ($10^{-6}$ M) resulted in modest but highly significant rises in perfusion pressure (Table 1). ET-3 ($10^{-8}$ M) also evoked a significant although much smaller pressor effect. ET-1 ($10^{-9}$ M) was again inactive in this respect (Table 1). Pre-treatment with either PD151242 or Ro 31-8220 significantly reduced the effects of both ET-1 ($10^{-8}$ M) and DOPPA ($10^{-6}$ M).

ET-1 ($10^{-8}$ M) was found to be less active than DOPPA when assessed for its ability to induce colloidal carbon leakage in villi microvessels (Table 2), although both compounds induced effects that were significantly greater than control values. Pre-treatment with Ro 31-8220 significantly reduced the effects of both ET-1 and DOPPA, but PD151242, while tending to reduce the effects of ET-1, only significantly reduced the effects of DOPPA (Table 2). ET-1 ($10^{-9}$ M) and ET-3 ($10^{-8}$ M) did not significantly affect the leakage of colloidal carbon (Table 2).

### Discussion

The three ET isopeptides that have been described,[3] act upon two ET receptors, $ET_A$ and $ET_B$.[17] $ET_A$ receptors have a higher affinity for ET-1 and ET-2 than for ET-3, whereas $ET_B$ receptors have a similar affinity for all three isopeptides. In arterial blood vessels activation of $ET_A$ receptors is associated with smooth muscle contraction, whereas activation of $ET_B$ receptors sometimes produces smooth muscle relaxation, the latter possibly via the release of nitric oxide.[18] Coupling of both types of ET receptor to phospholipase C via a G protein has been demonstrated.[19] However, in human and rabbit saphenous veins, stimulation of both $ET_A$ and $ET_B$ receptors is associated with smooth muscle contraction,[20,21] the $ET_A$-mediated response being PKC-dependent and the $ET_B$-mediated response being PKC-independent.[20]

The present results, using the mesenteric vessels, confirm other reports that ET-1 is a more potent vasoconstrictor than ET-3,[3, 21–23] and suggest that in isolated mesenteric arterioles the constrictor effect of ET-1 is mediated via $ET_A$ receptors, since it is abolished by pre-treatment with the specific $ET_A$ receptor antagonist PD151242, and significantly reduced by pre-treatment with the specific PKC inhibitor Ro 31-8220. The virtual lack of effect of the same concentration of ET-3 in this situation also suggests the absence of functionally important $ET_B$ receptors in mesenteric arterioles. Evidence from other workers indicates that $ET_A$ receptors are located mainly on the arterial side of the circulation and $ET_B$ receptors on the venous side,[24] although there are variations between species and organs.[23,24]

When the small intestine remains attached to the mesentery, capillaries and venules, as well as arterioles, are involved in the perfusion. In the light of work cited above[24] this would provide an opportunity for stimulation of $ET_B$ receptors, which might

**Table 1.** Effects of ET-1, ET-3 and DOPPA on perfusion pressures in rat isolated mesenteric and small intestinal vessels in the presence and absence of PD151242 ($10^{-6}$ M) or Ro 31-8220 ($10^{-6}$ M)

| Treatment | Alterations in perfusion pressure (mmHg) after 5 min perfusion[ab] | | | | | | |
|---|---|---|---|---|---|---|---|
| | Concentration (M) | *n* | GPSS | *n* | GPSS + PD151242 | *n* | GPSS + Ro 31-8220 |
| Control | – | 10 | −2.70 ± 0.90 | 3 | −2.33 ± 0.33 | 4 | −0.5 ± 1.19 |
| ET-1 | $10^{-8}$ | 7 | 32.71 ± 2.50[c] | 4 | 6.75 ± 3.33[d] | 4 | 3.75 ± 0.75[d] |
| ET-1 | $10^{-9}$ | 4 | 0.25 ± 0.85 | – | – | – | – |
| ET-3 | $10^{-8}$ | 5 | 2.80 ± 0.58[c] | – | – | – | – |
| DOPPA | $10^{-6}$ | 8 | 36.75 ± 7.34[c] | 5 | 14.80 ± 2.96[d] | 5 | 13.2 ± 2.69[d,e] |

[a]Perfusion pressure at the beginning of the 5 min perfusion period was taken as 0. [b] Results are mean ± S.E.M. [c]$p < 0.05$ (Bonferroni's test) compared with control values. [d]$p < 0.05$ (Bonferroni's test) compared with agonist alone. [e]Value taken from Reference 8.

**Table 2.** Leakage of colloidal carbon in microvessels of villi in the rat small intestine *in vitro* in response to ET-1, ET-3 or DOPPA, and the effects of pre-treatment with PD151242 ($10^{-6}$ M) or Ro 31-8220 ($10^{-6}$ M)

| Treatment | Amount of CC, determined by image analysis, assessed as % of frame area[ab] | | | | | | |
|---|---|---|---|---|---|---|---|
| | Concentration (M) | *n* | GPSS | *n* | GPSS + PD151242 | *n* | GPSS + Ro 31-8220 |
| Control | – | 10 | 1.05 ± 0.16 | 3 | 1.20 ± 0.08 | 4 | 0.83 ± 0.10 |
| ET-1 | $10^{-8}$ | 7 | 2.05 ± 0.28[c] | 4 | 139 ± 0.38 | 4 | 0.80 ± 0.11[d] |
| ET-1 | $10^{-9}$ | 4 | 1.62 ± 0.49 | – | – | – | – |
| ET-3 | $10^{-8}$ | 5 | 0.65 ± 0.11 | – | – | – | – |
| DOPPA | $10^{-6}$ | 8 | 3.74 ± 0.54[c] | 5 | 2.18 ± 0.24[d] | 5 | 0.90 ± 0.12[d,e] |

[a]Each frame represents one negative micrograph magnified approximately 20 times. [b]Results are given as mean ± S.E.M. [c]$p < 0.05$ (Bonferroni's test) compared with control value. [d]$p < 0.05$ (Bonferroni's test) compared with agonist alone. [e]Value taken from Reference 8.

then have a relaxant effect[18] in addition to the contracting effect brought about via arterial $ET_A$ receptors. Activation of two such opposing effects might explain the smaller rise in perfusion pressure brought about by ET-1 (32.75 ± 2.50 mmHg after 5 min of perfusion in the mesentery plus small intestine, compared with 85.80 ± 11.60 mmHg after 5 min perfusion in the mesentery alone). Alternatively, or in addition, differences in perfusion pressure rises in the two preparations may reflect differences in the flow rates of perfusate used.

PKC-dependent actions of ETs in native vascular smooth muscle[20,25,26] and in cultured vascular smooth muscle cells[27,28] have been demonstrated, as has a release of ETs by activation of PKC by phorbol esters.[11,12] Therefore, since the effects of DOPPA were reduced in both preparations used in the present work, by pre-treatment with either the $ET_A$ receptor antagonist or the PKC inhibitor, the possibility of a bi-directional link between these two processes arises. On the basis of present evidence, however, it is not possible to distinguish between mediation by ET-1 or ET-2, or by a mixture of ET-1 and ET-2, in these situations.

A similar link may also be involved in the DOPPA- and the ET-1 induced increases in vascular permeability that were manifest as a leakage of colloidal carbon into microvessel walls in small intestinal villi (Table 2). However, while ET-3 ($10^{-8}$ M) did not increase colloidal carbon leakage in the present experiments, other workers have shown that an application of ET-3 ($10^{-10}$ M) in conjunction with an $ET_A$ receptor antagonist increased microvascular permeability to FITC-labelled albumin in rat mesentery.[29] *In vivo*, the ET-1-induced increase in vascular permeability to Evans Blue dye appeared to be mediated via release of platelet-activating factor[30] but we have no information at the moment as to whether this also operated in our experimental situation. We have, in fact, shown that DOPPA-induced colloidal carbon leakage was enhanced by pre-treatment with indomethacin[13] suggesting a possible inhibitory effect of prostanoids.

Increases in microvascular permeability in response to various inflammatory agonists have been attributed to an increase in cytoplasmic $Ca^{2+}$ levels in Ecs, either as a result of influx from the extracellular medium or by release from intracellular stores.[31,32] Such increases in $Ca^{2+}$ levels have been demonstrated in response to bradykinin,[33,34] to platelet activating factor[35] and, more recently, to ET-1,[36,37] and are thought to lead to EC contraction. This results from an actomyosin shortening, brought about by phosphorylation of myosin light chains via myosin light chain kinase, which is a $Ca^{2+}$-calmodulin-dependent process.[38] Myosin light chains can also be phosphorylated by PKC,[39] as can the thin-filament regulatory protein caldesmon.[40] Therefore, previous reports showing that stimulation of ET-1 receptors induces a phosphorylation of myosin light chains and of caldesmon in arterial smooth muscle[41,42] are important in the present context, and support the suggested bi-directional link between activation of $ET_A$ receptors and activation of PKC.

### References

1. Yanagisawa M, Kurihara H, Kimura S, *et al.* A novel potent vasoconstrictor peptide produced by vascular endothelial cells. *Nature* 1988; **332**: 411–415.
2. Itoh Y, Yanagisawa M, Ohkubo S, *et al.* Cloning and sequence analysis of cDNA encoding the precursor of a human endothelium-derived vasoconstrictor peptide, endothelin: identity of human and porcine endothelin. *FEBS Lett* 1988; **231**: 440–444.
3. Inoue A, Yanagisawa M, Kimura S, *et al.* The human endothelin family: three structurally and pharmacologically distinct isopeptides predicted by three separate genes. *Proc Natl Acad Sci USA* 1989; **86**: 2863–2867.
4. Emori T, Hirata Y, Ohta K, *et al.* Cellular mechanism of endothelin-1 release by angiotensin and vasopressin. *Hypertension* 1991; **18**: 165–170.
5. Kuchan MJ, Frangos JA. Shear stress regulates endothelin-1 release via protein kinase C and cGMP in cultured endothelial cells. *Am J Physiol* 1993; **264**: H150–H156.
6. Wang DL, Tang CC, Wung BS, Chen HH, Hung MS, Wang JJ. Cyclical strain increases endothelin-1 secretion and gene expression in human endothelial cells. *Biochem Biophys Res Comm* 1993; **195**: 1050–1056.
7. Northover AM, Northover BJ. Sapintoxin A and phorbol 12,13-dibutyrate: two phorbol derivatives with contrasting effects on rat blood vessels *in vitro*. *J Pharm Pharmacol* (in press).
8. Northover AM, Northover BJ. Vasoconstriction in rat isolated mesentery and small intestine in response to various activators of protein kinase C. *Agents Actions* (in press).
9. Kikkawa U, Takai Y, Tanaka Y, Miyake R, Nishizuka Y. Protein kinase C as a possible receptor protein of tumor-promoting phorbol esters. *J Biol Chem* 1983; **258**: 11442–11445.
10. Abdel-Latif AA. Calcium-mobilizing receptors, polyphosphoinositides, and the generation of second messengers. *Pharmacol Rev* 1986; **38**: 227–272.
11. Yanagisawa M, Inoue A, Takuwa Y, Mitsui Y, Kobayashi M, Masaki T. The human preproendothelin-1 gene: possible regulation by endothelial phosphoinositide turnover signaling. *J Cardiovasc Pharmacol* 1989; **13** (Suppl 5): S13–S17.
12. Inoue A, Yanagisawa M, Takuwa Y, Mitsui Y, Kobayashi M, Masaki T. The human preproendothelin-1 gene. Complete nucleotide sequence and regulation of expression. *J Biol Chem* 1989; **264**: 14954–14959.
13. Northover AM, Northover BJ. Stimulation of protein kinase C activity may increase microvascular permeability to colloidal carbon via α-isoenzyme. *Inflammation* 1994; **18**: 481–487.
14. Davenport AP, Kuc RE, Fitzgerald F, Maguire JJ, Berryman K, Doherty AM. [$^{125}$I]-PD151242: a selective radioligand for human $ET_A$ receptors. *Br J Pharmacol* 1994; **111**: 4–6.
15. Davis PD, Hill CH, Keech E, *et al.* Potent selective inhibitors of protein kinase C. *FEBS Lett* 1989; **259**: 61–63.
16. Northover AM. An *in vitro* method for assessing the effects of pro-inflammatory and anti-inflammatory compounds on microvascular permeability in the rat small intestine. *J Pharmacol Toxicol Meth* 1993; **29**: 227–232.
17. Sakurai T, Yanagisawa M, Takuwa Y, *et al.* Cloning of a cDNA encoding a non-isopeptide-selective subtype of the endothelin receptor. *Nature* 1990; **348**: 732–735.
18. Sudjarwo SA, Hori M, Karaki H. Effect of endothelin-3 on cytosolic calcium level in vascular endothelium and on smooth muscle contraction. *Eur J Pharmacol* 1992; **229**: 137–142.
19. Masaki T, Vane JR, Vanhoutte PM. International union of pharmacology nomenclature of endothelin receptors. *Pharmacol Rev* 1994; **46**: 137–142.
20. Gray GA, Löffler B-M, Clozel M. Characterization of endothelin receptors mediating contraction of rabbit saphenous vein. *Am J Physiol* 1994; **266**: H959–H966.
21. White DG, Garratt H, Mundin JW, Sumner MJ, Vallance PJ, Watts IS. Human saphenous vein contains both endothelin $ET_A$ and $ET_B$ contractile receptors. *Eur J Pharmacol* 1994; **257**: 307–310.
22. Wallace JL, Keenan CM, MacNaughton WK, McKnight GW. Comparison of the effects of endothelin-1 and endothelin-3 on the rat stomach. *Eur J Pharmacol* 1989; **167**: 41–47.
23. Maguire JJ, Kuc RE, O'Reilly G, Davenport AP. Vasoconstrictor endothelin receptors characterized in human renal artery and vein *in vitro*. *Br J Pharmacol* 1994; **113**: 49–54.
24. Moreland S, McMullen D, Abboa-Offei B, Seymour A. Evidence for a differential location of vasoconstrictor endothelin receptors in the vasculature. *Br J Pharmacol* 1994; **112**: 704–708.
25. Auguet M, Delaflotte S, Chabrier PE, Braquet P. Comparative effects of endothelin and phorbol 12-13 dibutyrate in rat aorta. *Life Sci* 1989; **45**: 2051–2059.
26. Nishimura J, Moreland S, Ahn HY, Kawase T, Moreland RS, van Breemen C. Endothelin increases myofilament $Ca^{2+}$ sensitivity in α-toxin-permeabilized rabbit mesenteric artery. *Circ Res* 1992; **71**: 951–959.
27. Griendling KK, Tsuda T, Alexander RW. Endothelin stimulates diacylglycerol accumulation and activates protein kinase C in cultured vascular smooth muscle cells. *J Biol Chem* 1989; **264**: 8237–8240.

28. Danthuluri NR, Brock TA. Endothelin receptor-coupling mechanisms in vascular smooth muscle: a role for protein kinase C. *J Pharmacol Exp Ther* 1990; **254**: 393–399.
29. Kurose I, Miura S, Fukumura D, Tsuchiya M. Mechanisms of endothelin-induced macromolecular leakage in microvascular beds of rat mesentery. *Eur J Pharmacol* 1993; **250**: 85–94.
30. Filep JG, Sirois MG, Rousseau A, Fournier A, Sirois P. Effects of endothelin-1 on vascular permeability in the conscious rat: interactions with platelet-activating factor. *Br J Pharmacol* 1991; **104**: 797–804.
31. Northover AM. Action of histamine on endothelial cells of guinea-pig isolated hepatic portal vein and its modification by indomethacin or removal of calcium. *Br J Exp Path* 1975; **56**: 52–61.
32. Northover AM. The effects of TMB-8 on the shape changes of vascular endothelial cells resulting from exposure to various inflammatory agents. *Agents Actions* 1989; **26**: 367–371.
33. Colden-Stanfield M, Schilling WP, Ritchie AK, Eskin SG, Navarro LT, Kunze DL. Bradykinin-induced increases in cytosolic calcium and ionic currents in cultured bovine aortic endothelial cells. *Circ Res* 1987; **61**: 632–640.
34. Morgan-Boyd R, Stewart JM, Vavrek RJ, Hassid A. Effects of bradykinin and angiotensin II on intracellular $Ca^{2+}$ dynamics in endothelial cells. *Am J Physiol* 1987; **253**: C588–C598.
35. Hirafuji M, Maeyama K, Watanabe T, Ogura Y. Transient increase of cytosolic free calcium in cultured human vascular endothelial cells by platelet-activating factor. *Biochem Biphys Res Comm* 1988; **154**: 910–917.
36. Yokokawa K, Kohno M, Murakawa K, Yasunari K, Takeda T. Effect of endothelin-1 on cytosolic calcium ions in cultured human endothelial cells. *J Hypertension* 1990; **8**: 843–849.
37. Aoki H, Kobayashi S, Nishimura J, Kanaide H. Sensitivity of G-protein involved in endothelin-1-induced $Ca^{2+}$ influx to pertussis toxin in porcine endothelial cells *in situ*. *Br J Pharmacol* 1994; **111**: 989–996.
38. Kamm KE, Stull JT. The function of myosin and myosin light chain kinase phosphorylation in smooth muscle. *Ann Rev Pharmacol Toxicol* 1985; **25**: 593–620.
39. Ikebe M, Inagaki M, Kanamaru K, Hidaka H. Phosphorylation of smooth muscle myosin light chain kinase by $Ca^{2+}$-activated, phospholipid-dependent protein kinase. *J Biol Chem* 1985; **260**: 4547–4550.
40. Umekawa H, Hidaka H. Phosphorylation of caldesmon by protein kinase C. *Biochem Biophy Res Comm* 1985; **132**: 56–62.
41. Adam LP, Milio L, Brengle B, Hathaway DR. Myosin light chain and caldesmon phosphorylation in arterial muscle stimulated with endothelin-1. *J Mol Cell Cardiol* 1990; **22**: 1017–1023.
42. Abe Y, Kasuya Y, Kudo M, *et al.* Endothelin-1-induced phosphorylation of the 20-kDa myosin light chain and caldesmon in porcine coronary artery smooth muscle. *Japan J Pharmacol* 1991; **57**: 431–435.

**Received 7 September 1994; accepted in revised form 14 October 1994**
